# Beyond silent scans: voice assistants and the future of diagnostic imaging

**DOI:** 10.1007/s00330-024-11200-w

**Published:** 2024-11-15

**Authors:** Matthias A. Fink

**Affiliations:** 1 Clinic for Diagnostic and Interventional Radiology, University Hospital Heidelberg, Heidelberg, Germany; 2 Translational Lung Research Center Heidelberg, Member of the German Center for Lung Research, Heidelberg, Germany

Imagine being able to command your entire radiology workflow with your voice. “Alexa, pull up the last chest CT,” or “Hey Siri, create a summary of the patient’s history.” It may sound like science fiction, but this is where radiology could be heading as voice assistants (VAs) become increasingly integrated into healthcare [1]. While VAs are already transforming retail by turning casual interactions into purchase decisions [2, 3], radiology could eventually adopt similar technology to transform diagnostic imaging workflows.

We are already familiar with the concept of asking VAs about the weather or requesting a song. But using them to streamline the complex and demanding process of medical imaging? This is the next frontier. Advances in natural language processing (NLP) and artificial intelligence (AI) are transforming VAs from mere gadgets into powerful tools that can potentially transform the efficiency and accuracy of radiology workflows. However, as with retail, the adoption of this technology is not without its challenges—trust, perceived usefulness, and privacy concerns remain key barriers that need to be addressed. To understand the potential adoption of VAs in radiology, we can turn to the technology acceptance model (TAM), a framework widely used in retail contexts [4]. The TAM posits that the perceived usefulness and ease of use of technology are key determinants of its adoption. In the context of radiology, this means that VAs must be proven to improve workflow efficiency and be intuitive to use for radiologists to adopt them. As with any new technology, radiologists will not immediately hand over critical tasks such as reporting or image analysis to a machine unless they are confident in its capabilities. A key factor in this confidence is perceived usefulness—whether the VA will actually make life easier by assisting with tasks that reduce cognitive load and increase productivity. Much like the retail sector, where consumers are attracted to VAs that help them find the perfect gift, radiologists need to experience first-hand how VAs can improve their workflow, whether it is quickly retrieving patient data or dictating complex reports in real-time.

The social role of a VA will also play a key role in its acceptance. Research shows that users are more likely to trust and engage with VAs that take on a more personable role—more like a colleague offering advice rather than a robotic tool following orders [5]. In radiology, a VA that can understand the nuances of imaging terminology and anticipate the radiologist’s needs could become a trusted companion in the diagnostic process, rather than just another machine to operate. This aspect of personalisation is crucial. Just as retail VAs aim to understand a user’s shopping preferences, a radiology VA could learn a particular radiologist’s reporting style, preferred image viewing settings, or commonly used reference materials. The goal is to create a symbiotic relationship in which the VA enhances the radiologist’s capabilities, rather than attempting to replace human expertise.

However, in the healthcare sector, where sensitive patient data is at stake, privacy is a major concern. The personalisation-privacy paradox observed in retail [6] is no different in radiology. While VAs offer the allure of personalised support and tailored workflows, the exchange of personal data—especially in a field as sensitive as medicine—raises questions about security and trust. Radiologists need to be reassured that patient data will be handled with the utmost care before fully integrating VAs into their daily practice. This paradox is a unique challenge in healthcare. On the one hand, the more data a VA has access to, the more helpful it can be in providing contextual assistance. On the other hand, the privacy and security of patient information is important. Finding the right balance will be critical to the successful implementation of VAs in radiology.

Another important consideration is the potential for habit formation. As radiologists become more comfortable with VA-assisted workflows, these interactions may become automatic, much like how consumers may habitually add items to their shopping carts via voice commands without much thought. While this can lead to increased efficiency, it also raises concerns about over-reliance on technology and the potential for reduced vigilance in critical decision-making processes. This phenomenon is known as automation bias, where humans tend to rely too heavily on automated systems, potentially leading to errors if the system is incorrect or misleading [7]. In the context of radiology, where every decision can have a significant impact on health, it is important to strike a balance between using the convenience of VAs and preserving the critical thinking and expertise of radiologists. The aim should be to use VAs as enhancers of human skills rather than replacements for human judgement.

As we begin to explore the potential of voice-enabled workflows in radiology, it is critical to address these concerns head-on. Only then can we begin to realise the true potential of VAs in transforming diagnostic imaging—transforming the technology from just another gadget to an indispensable tool that improves the accuracy and efficiency of radiology practice. Several steps are needed to achieve this. We need to develop VAs that are specifically tailored to radiology workflows, ensuring that they understand medical terminology and can interface seamlessly with existing picture archiving and communication (PACS) and radiology information (RIS) systems. It is important to implement robust privacy and security measures that comply with healthcare regulations while still allowing for personalised assistance.

Extensive usability testing with radiologists is essential to refine the interface and capabilities of the VA to ensure that it enhances rather than hinders workflow. Equally important is the implementation of comprehensive training programmes for radiologists to enable them to use VAs effectively while maintaining their critical thinking skills and professional judgement. It is also important to establish clear guidelines for the appropriate use of VAs in radiology. These guidelines should define when human oversight is essential and provide protocols for resolving discrepancies between VA suggestions and radiologist opinions.

By considering these theoretical frameworks and addressing the unique challenges of the healthcare environment, we can harness the potential of VAs to drive greater efficiency and precision in diagnostic imaging. The future of radiology is not just about silent scans—it’s about giving voice to a more intuitive, responsive and effective diagnostic process.
